# Assessment of the clinical utility of the Gail model in estimating the risk of breast cancer in women from the Indian population

**DOI:** 10.3332/ecancer.2013.363

**Published:** 2013-10-17

**Authors:** Vasu Reddy Challa, Krishnamurthy Swamyvelu, Naren Shetty

**Affiliations:** Kidwai Memorial Institute of Oncology, Bengaluru, Karnataka 560029, India

**Keywords:** breast cancer, Gail model, risk assessment models, breast cancer screening

## Abstract

**Introduction:**

Breast cancer screening programmes are based on various risk models to assess the risk of breast cancer in the general population. The aim of the present study is to predict the efficacy of the Gail model (GM) in the Indian population. We did a retrospective calculation of the Gail score from the hospital records of patients with breast cancer and benign breast disease.

**Materials and methods:**

The Gail score was calculated in three groups. The three groups were made up of 104 patients with confirmed breast cancer (Group A), 100 patients with confirmed benign breast diseases (Group B), and 100 patient attendants (Group C).

**Statistical analysis:**

The data analysis was done using SPSS 15.0, Medcal 9.0.1.

**Results:**

The median Gail score in the three groups of patients was 7.5±3.04 in patients with breast cancer, 8.2±1.4 in patients with benign breast diseases, and 7.8±1.7 in normal people. The median Gail score was lower in patients with breast cancer when compared with normal people.

**Conclusion:**

The GM is not useful in identifying the risk of breast cancer in Indian women. There is a need for further studies to evaluate other genetic and environmental factors to create an appropriate model for the Indian population.

## Introduction

The incidence of breast cancer in India is constantly increasing due to late marriage, fewer children, changes in dietary habits, and increased detection rate because of increased public awareness. Currently, India reports about 100,000 cases of breast cancer annually, but there is an estimated increased rate of 3% per year with an estimated 250,000 cases per annum by 2015. The estimated rate of breast cancer in India is 80 new cases per 100,000 population per year, and in Delhi, it is estimated to be 146 new cases per 100,000 population per year [1]. This highlights the importance of breast cancer screening programmes in India.

Gail et al developed a model for estimating the risk of developing breast cancer from the case-control data of the Breast Cancer Detection Demonstration Project (BCDDP) (Table 1) [2]. This was called Gail model 1 (GM 1), and it estimated the probability of invasive ductal carcinoma, ductal carcinoma *in situ*, and lobular carcinoma *in situ*. GM 2 was modified by statisticians of the National Surgical Adjuvant Breast and Bowel Project (NSABP) to project the risk of developing invasive breast cancer only to determine eligibility for the breast cancer prevention trial, which is available on the National Cancer Institute (NCI) website [3]. The major drawback of the GM is it includes only first-degree relatives, which results in underestimating the risk in 50% of families who have cancer in the paternal lineage [4]. Various other models were used in different studies such as the Claus model, which was developed to assess familial risk of breast cancer from a case-control study done by the Centers for Disease control. Parmigiani and colleagues developed BRCAPRO model where hereditary factors were considered mainly in assessing risk of BRCA 1 and BRCA 2 mutations in a family [5]. The Tyrer–Cuzick model is the only model that incorporates multiple epigenetic factors and detailed family history for assessing risk and was used as an alternative for the GM for eligibility for the International Breast Intervention Study (IBIS-1) [6].

The GM is the most commonly used risk prediction model and has been well studied and applied in various studies. Here, we would like to apply the GM to the Indian population and assess whether it can be applied to assess the prediction of breast cancer for the Indian population.

## Materials and methods

This was a retrospective study of patients treated in a single unit between January 2011 and March 2012. Group A consisted of 104 women above 35 years with invasive breast cancer who had undergone treatment in a single unit at a tertiary care cancer centre. A cohort of 100 patients who underwent surgery for benign breast diseases at our centre were randomly selected (Group B), and another control cohort of 100 female patient attendants above 35 years with no personal or family history of breast cancer or ovarian cancer were included in the study (Group C). Informed consent was taken from all the study subjects.

The risk factors used were age at menarche, age at first live birth, previous biopsy (number and atypical hyperplasia), and number of first-degree relatives with a history of breast cancer. The calculation was done by using GM prediction from the NCI’s breast cancer Risk Assessment Tool website (which is available at http://www.cancer.gov/bcrisktool/).

**Statistics: **Statistical software, namely, SPSS 15.0 and MedCalc 9.0.1, was used to perform descriptive statistics. Median, standard error, and Kruskal–Wallis test were performed using MedCalc software.

## Results

The Gail scores for lifetime risk of cancer were calculated for 104 patients with proven breast cancer (Group A), 100 patients with benign breast diseases (Group B), and 100 normal controls (Group C). Of 100 patients with benign breast disease, 21 had fibroadenoma, 35 had fibrocystic disease, 29 had periductal mastitis/duct ectasia, eight patients had granulomatous mastitis, five patients had phyllodes tumour, and two patients had fat necrosis. The mean age distribution of the patients in the three groups is described in Table 2. The age at menarche distribution showed the difference among Group A, Group B, and Group C (Table 3). Four patients underwent benign biopsy in Group A and one patient in Group B had a history of previous biopsy. Among four patients in Group A, three had a biopsy for atypical ductal hyperplasia (ADH) and one for atypical lobular hyperplasia, and in Group B, one had a previous biopsy for ADH. Seven patients in Group A and one patient in group B had a history of a first-degree relative with breast cancer. Considering another risk factor, age at first live birth, nine patients had experienced child birth after 30 years of age in Group A with none in Group B and Group C (Table 4). A history of the oral contraceptive pill was present in two patients of Group A both with a duration of 1 and 2 years. We also studied the number of children in the three groups that showed 14 nulliparous women in Group A, 4 in Group B, and none in Group C (Table 5).

**Figure 1.**
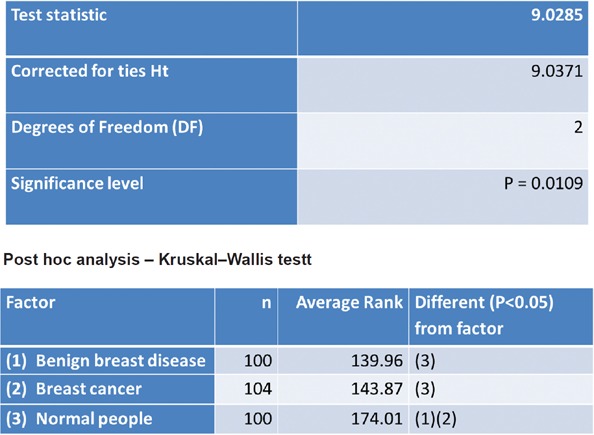
Kruskal–Wallis test.

**Figure 2.**
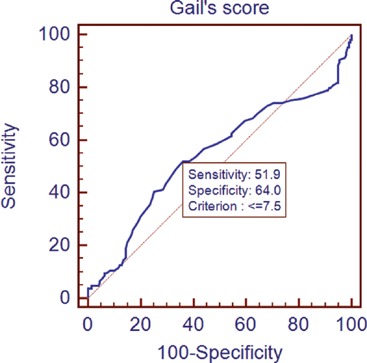
Receiver operating characteristic (ROC) curves plotting true positive (sensitivity) versus false-positive fraction (100-specificity).

The lifetime risk of breast cancer was calculated using the GM (Table 6). A non-parametric test using the Kruskal–Wallis test showed a significant difference in the Gail scores among the three groups (Figure 1). Further post-hoc analysis showed significantly higher levels of Gail score in normal people compared with benign breast disease and breast cancer patients. The receiver operating characteristic (ROC) curve has been used to evaluate the diagnostic accuracy of the GM. The area under the curve was 0.543. At a Gail score of 7.5, the sensitivity and specificity of GM were 51.9% and 64%, respectively (Figure 2).

## Discussion

Most Western countries use the GM to assess the risk of breast cancer, and recently, European countries have been using the Tyrer–Cuzick model. As the incidence of breast cancer is rising in India, it is important to detect patients with a high risk of breast cancer for timely treatment. Mitchell Gail, a biostatistician, developed a mathematical model in 1989 to assess the risk of breast cancer risk based on the results from the BCDDP–a large screening study that included 284,780 women who had been undergoing annual mammographic examination [2]. Later, it was modified by involving atypical hyperplasia in breast biopsy, race, and ethnicity [3]. The drawbacks of the GM were that it does not consider age at diagnosis of breast cancer, lobular neoplasia, family history of breast cancer in second-degree relatives, and family history of ovarian cancer. This led to the development of various other models considering the factors that were neglected in the GM such as history of breast cancer in second-degree relatives, which was included in the Tyrer–Cuzick model.

The other countries and cities who validated the GM apart from the United States are Canada [7], Edinburgh [8], Malmo [9, 10], Kopparberg and Ostergotland (Swedish Two-County) [11], Stockholm [12], Gothenburg [10, 13] and Turkey [14], and Singapore [15]. These studies found a difficulty in including the Asian population in their studies. Recently, a Singapore breast cancer screening project attempted to validate the GM in a prospective nationwide study with screening mammograms for 28,104 women. They concluded that the GM overestimates invasive breast cancer in an Asian developed nation especially in the age group of 60–64 years [15]. Another study reports on the development of the Asian American Breast Cancer Study (AABCS) model to estimate ethnicity-specific absolute risks for breast cancer in Asian and Pacific Islander American (APA) women living in the United States [16].

There are no studies in India to date in assessing predictive breast cancer risk models. In our study, the Gail score for patients with breast cancer was lower than for normal people. The study is limited by its small size. Further studies with a larger percentage of the population are required to validate the GM in a prospective manner including the race-specific incidence rates of invasive breast cancer and other causes of mortality.

## Conclusion

We should be cautious in using the GM as it has not been verified in the Indian population. More risk factors need to be evaluated in our population; age-specific incidence rates of breast cancer need to be included in the GM and prospectively validated in our population. This may help in the development of future screening programmes in India.

## Figures and Tables

**Table 1.** Various risk assessment models and the factors considered in assessing the risk of breast cancer.

Gail model	Claus model	BRCAPRO model	Tyrer–Cuzick model	BOADICEA model
Age of the person Age at menarche Age at first live birth Breast biopsies (AH) Family history - First-degree relatives	Age of the person Age at menarche Age at first live birth Family history - First-degree relatives - Second-degree relatives	Age of the person Family history - First-degree relatives - Second-degree relatives - Third-degree relatives - Age at onset of breast cancer - Bilateral breast cancer - Ovarian cancer - Male breast cancer	Age of the person Body mass index Age at menarche Age at first live birth Age at menopause Hormone replacement therapy use Breast biopsies (ADH, LCIS) Family history - First-degree relatives - Second-degree relatives - Age at onset of breast cancer - Bilateral breast cancer - Ovarian cancer	Age of the person Family history - First-degree relatives - Second-degree relatives - Third-degree relatives - Age at onset of breast cancer - Bilateral breast cancer - Ovarian cancer - Male breast cancer

AH, atypical hyperplasia; LCIS, lobular carcinoma *in situ;* BOADICEA, breast and ovarian analysis of disease incidence and carrier estimation algorithm.

**Table 2.** Age distribution of patients studied.

Age in years	Group A		Group B		Group C	
	No.	%	No.	%	No.	%
<40	19	18.2	32	32.0	29	29.0
40–50	35	33.6	42	42.0	26	26.0
50–60	28	29.0	22	22.0	21	21.0
60–70	16	15.2	3	3.0	23	23.0
>70	6	5.0	1	1.0	1	1.0
Total	104	100.0	100	100.0	100	100.0
Median ±SE	48.5±1.05		42 ±0.7		45.0±1.0	

**Table 3.** Age at menarche distribution of patients studied.

Age at menarche in years	Group A		Group B		Group C	
	No.	%	No.	%	No.	%
≤11	6	5.8	4	4.0	21	21.0
12–13	47	45.2	48	48.0	61	61.0
≥14	51	49.0	48	48.0	18	18.0
Total	104	100.0	100	100.0	100	100.0
Median ±SE	13.0±0.1		13.0±0.1		12.0±0.1	

**Table 4.** Age at first live birth.

Age at first live birth	Group A		Group B		Group C	
	No.	%	No.	%	No.	%
Nil	14	13.4	3	3.0	0	0.0
<20	36	34.6	13	13.0	34	34.0
20–24	32	30.8	78	78.0	62	62.0
25–29	13	12.5	6	6.0	4	4.0
30 and above	9	8.7	0	0.0	0	0.0
Total	104	100.0	100	100.0	100	100.0
Median ±SE	20±0.8		21±0.4		21±0.2	

**Table 5.** Number of children of subjects in three groups studied.

Number of children	Group A		Group B		Group C	
	No.	%	No.	%	No.	%
Nil	14	13.4	4	4.0	0	0.0
1–2	37	35.5	73	73.0	54	54.0
3–4	45	43.2	20	20.0	40	40.0
5 and above	8	7.6	3	3.0	6	6.0
Total	104	100.0	100	100.0	100	100.0
Median ±SE	3±0.1		2±0.1		2±0.1	

**Table 6.** Gail’s life time risk of cancer.

Gail’s life time risk of cancer	Group A		Group B		Group C	
	No.	%	No.	%	No.	%
<5.0	8	7.6	2	2.0	9	9.0
5–10	75	72.1	91	91.0	84	84.0
10–20	17	16.3	7	7.0	7	7.0
>20	2	1.9	0	0.0	0	0.0
Total	104	100.0	100	100.0	100	100.0
Median ±SE	7.5±0.3		8.2±0.1		7.8±0.1	

SE, standard error.

## References

[1] Bagchi S (2008). Breast cancer rises in India. CMAJ.

[2] Gail M, Brinton L, Byar D (1989). Projecting individualized probabilities of developing breast cancer for white females who are being examined annually. J Natl Cancer Inst.

[3] Costantino J, Gail M, Pee D (1999). Validation studies for models projecting the risk of invasive and total breast cancer incidence. J Natl Cancer Inst.

[4] Gareth D, Evans R, Anthony Howell (2007). Breast cancer risk-assessment models. Breast Cancer Res.

[5] Parmigiani G, Berry DA, Aquilar O (1998). Determining carrier probabilities for breast cancer susceptibility genes BRCA1 and BRCA2. Am J Hum Genet.

[6] Tyrer J, Duffy SW, Cuzick J (2004). A breast cancer prediction model incorporating familial and personal risk factors. Stat Med.

[7] Miller AB, To T, Baines CJ, Wall C (2000). Canadian National Breast Screening Study-2: 13-year results of a randomized trial in women aged 50-59 years. J Natl Cancer Inst.

[8] Alexander FE, Anderson TJ, Brown HK, Forrest AP, Hepburn W, Kirkpatrick AE (1999). 14 years of follow-up from the Edinburgh randomised trial of breast-cancer screening. Lancet.

[9] Andersson I, Aspegren K, Janzon L, Landberg T, Lindholm K, Linell F (1998). Mammographic screening and mortality from breast cancer: the Malmo mammographic screening trial. BMJ.

[10] Nystrom L, Rutqvist LE, Wall S, Lindgren A, Lindqvist M, Ryden S (1993). Breast cancer screening with mammography: overview of Swedish randomized trials. Lancet.

[11] Tabar L, Fagerberg G, Chen HH, Duffy SW, Smart CR, Gad A (1995). Efficacy of breast cancer screening by age. New results from the Swedish Two-County Trial. Cancer.

[12] Frisell J, Lidbrink E, Hellstrom L, Rutqvist LE (1997). Followup after 11 years– update of mortality results in the Stockholm mammographic screening trial. Breast Cancer Res Treat.

[13] Bjurstam N, Bjorneld L, Duffy SW, Smith TC, Cahlin E, Eriksson O (1997). The Gothenburg breast screening trial: first results on mortality, incidence, and mode of detection for women ages 39-49 years at randomization. Cancer.

[14] Ulusoy C, Kepenekci I, Kose K, Aydintug S, Cam R (2010). Applicability of the Gail model for breast cancer risk assessment in Turkish female population and evaluation of breastfeeding as a risk factor. Breast Cancer Res Treat.

[15] Chay WY, Ong WS, Tan PH, Jie Leo NQ, Ho GH, Wong CS (2012). Validation of the Gail model for predicting individual breast cancer risk in a prospective nationwide study of 28,104 Singapore women. Breast Cancer Res.

[16] Matsuno RK, Costantino JP, Ziegler RG, Anderson GL, Li H, Pee D, Gail MH (2011). Projecting individualized absolute invasive breast cancer risk in Asian and Pacific Islander American women. J Natl Cancer Inst.

